# Glial cell response and microthrombosis in aneurysmal subarachnoid hemorrhage patients: An autopsy study

**DOI:** 10.1093/jnen/nlad050

**Published:** 2023-07-21

**Authors:** Inez Koopman, Bart J van Dijk, Nicolaas P A Zuithoff, Jacqueline A Sluijs, Marije J van der Kamp, Zelonna A V Baldew, Catharina J M Frijns, Gabriel J E Rinkel, Elly M Hol, Mervyn D I Vergouwen

**Affiliations:** Department of Neurology and Neurosurgery, UMC Utrecht Brain Center, University Medical Center Utrecht, Utrecht University, Utrecht, the Netherlands; Department of Translational Neuroscience, UMC Utrecht Brain Center, University Medical Center Utrecht, Utrecht University, Utrecht, the Netherlands; Julius Center for Health Sciences and Primary Care, University Medical Center Utrecht, Utrecht University, Utrecht, the Netherlands; Department of Translational Neuroscience, UMC Utrecht Brain Center, University Medical Center Utrecht, Utrecht University, Utrecht, the Netherlands; Department of Translational Neuroscience, UMC Utrecht Brain Center, University Medical Center Utrecht, Utrecht University, Utrecht, the Netherlands; Department of Translational Neuroscience, UMC Utrecht Brain Center, University Medical Center Utrecht, Utrecht University, Utrecht, the Netherlands; Department of Neurology and Neurosurgery, UMC Utrecht Brain Center, University Medical Center Utrecht, Utrecht University, Utrecht, the Netherlands; Department of Neurology and Neurosurgery, UMC Utrecht Brain Center, University Medical Center Utrecht, Utrecht University, Utrecht, the Netherlands; Department of Translational Neuroscience, UMC Utrecht Brain Center, University Medical Center Utrecht, Utrecht University, Utrecht, the Netherlands; Department of Neurology and Neurosurgery, UMC Utrecht Brain Center, University Medical Center Utrecht, Utrecht University, Utrecht, the Netherlands

**Keywords:** Astrocytes, Autopsy, Brain aneurysm, Glia, Inflammation, Microglia, Microthrombosis, Subarachnoid hemorrhage

## Abstract

Neuroinflammation and microthrombosis may be underlying mechanisms of brain injury after aneurysmal subarachnoid hemorrhage (aSAH), but they have not been studied in relation to each other. In postmortem brain tissue, we investigated neuroinflammation by studying the microglial and astrocyte response in the frontal cortex of 11 aSAH and 10 control patients. In a second study, we investigated the correlation between microthrombosis and microglia by studying the microglial surface area around vessels with and without microthrombosis in the frontal cortex and hippocampus of 8 other aSAH patients. In comparison with controls, we found increased numbers of microglia (mean ± SEM 50 ± 8 vs 20 ± 5 per 0.0026 mm³, p < 0.01), an increased surface area (%) of microglia (mean ± SEM 4.2 ± 0.6 vs 2.2 ± 0.4, p < 0.05), a higher intensity of the astrocytic intermediate filament protein glial fibrillary acidic protein (GFAP) (mean ± SEM 184 ± 28 vs 92 ± 23 arbitrary units, p < 0.05), and an increased GFAP surface area (%) (mean ± SEM 21.2 ± 2.6 vs 10.7 ± 2.1, p < 0.01) in aSAH tissue. Microglia surface area was approximately 40% larger around vessels with microthrombosis than those without microthrombosis (estimated marginal means [95% CI]; 6.1 [5.4–6.9] vs 4.3 [3.6–5.0], p < 0.001). Our results show that the microglial and astrocyte surface areas increased after aSAH and that microthrombosis and microglia are interrelated.

## INTRODUCTION

Aneurysmal subarachnoid hemorrhage (aSAH) is a type of stroke associated with high morbidity and fatality (1, 2). Neuroinflammation and microthrombosis may be underlying mechanisms of brain injury after aSAH (3, 4). Neuroinflammation is mediated by microglia, the resident immune cells of the brain, and astrocytes (5, 6). Previous studies in experimental ischemic stroke and intracerebral hemorrhage have shown activation of microglia and astrocytes after the stroke (7–14). In a previous study in human SAH patients microglia were activated following hemorrhage (6), but the astrocytic response after SAH has only been reported in animal studies (10–12). In addition, neuroinflammation and microthrombosis have been described as separate processes (6, 15, 16), but they have not been studied in relation to each other. Knowledge about this correlation can provide more insight into the mechanisms underlying brain injury associated with aSAH.

In this study, we first investigated astrocyte and microglial responses in human aSAH postmortem material. We hypothesized that these glial cells would show increased surface areas, based on validated markers (17, 18). Secondly, we hypothesized that microglial surface area is increased around vessels with microthrombosis and therefore studied the correlation between microthrombi and microglia by determining microglial surface area around vessels with and without microthrombosis.

## MATERIALS AND METHODS

We used postmortem brain tissue of aSAH patients, which consisted of residual hospital autopsy tissue obtained from the pathology department of the UMC Utrecht. The Biobank Research Ethics Committee of the UMC Utrecht (UMC Utrecht, 19-096) approved the use of residual postmortem autopsy tissue of aSAH patients for this study. Postmortem brain tissue of age-matched controls without a neurological disorder was provided by the Netherlands Brain Bank and was obtained based on informed consent of the brain donor for tissue use and accessing neuropathological and clinical information for scientific research.

### Microglia and astrocyte response

#### Human postmortem brain tissue

In the medial frontal gyrus of 11 aSAH patients and 10 age-matched controls without a neurological disorder, we assessed the glial cell response based on validated markers (Tables 1 and 2; Supplementary Data  Table S1). Some sections were taken from brain areas in close vicinity of the ruptured aneurysm (Supplementary Data  Tables S1 and S3). However, we did not take brain sections that were in close vicinity of an intraparenchymal hemorrhage. All aSAH patients had died within 20 days after the hemorrhage.

**Table 1.** Characteristics of aSAH patients

Microglia and astrocyte response	n = 11 (%)
Female sex	9 (82)
Mean age (SD)	69 (11)
Anterior circulation aneurysm	91 (10)
Posterior circulation aneurysm	1 (9)
Cause of death:	
-Initial aSAH	2 (18)
-Rebleeding	7 (64)
-Delayed cerebral ischemia	1 (9)
-Pneumonia	1 (9)
Median day of death after ictus [IQR]	8 [1–17]

**Microthrombosis and microglia surface area**	n = 8 (%)

Female sex	5 (63)
Mean age (SD)	61 (19)
Anterior circulation aneurysm	6 (75)
Posterior circulation aneurysm	1 (13)
Anterior and posterior circulation aneurysm	1 (13)
Cause of death:	
-Initial aSAH	4 (50)
-Rebleeding	2 (25)
-Delayed cerebral ischemia	1 (13)
-Pneumonia	1 (13)
Median day of death after ictus [IQR]	1 [0.3–9]

Aneurysm location is specified for the ruptured aneurysm. aSAH: aneurysmal subarachnoid hemorrhage; BA: basilar artery; Acom: anterior communicating artery; MCA: middle cerebral artery; ICA: internal carotid artery; VA: vertebral artery; Pcom: posterior communicating artery.

**Table 2.** Characteristics of age-matched controls without a neurological disorder

Number	Sex	Age	Cause of death	Braak score	Amyloid	PMD (h:min)	pH CSF
1	Female	47	Malignancy	1	0	04:00	6.9
2	Female	53	Respiratory failure	0	0	07:25	Unknown
3	Female	60	Malignancy	0	0	08:10	6.6
4	Female	72	Malignancy	1	A	06:50	7.2
5	Female	76	Malignancy	2	0	07:15	6.9
6	Female	77	Renal failure	1	A	08:20	6.5
7	Female	84	Malignancy	1	0	06:55	Unknown
8	Male	49	Malignancy	0	0	06:15	6.2
9	Male	56	Unknown	0	0	14:00	7.0
10	Male	80	Renal failure	0	0	07:15	5.8

PMD: postmortem delay of obduction; CSF: cerebrospinal fluid; Braak score: tau score for Alzheimer pathology; Amyloid: score for amyloid plaque pathology.

#### Immunohistochemistry

Formalin-fixed paraffin-embedded human brain tissue was cut in 7-µm sections, mounted on super frost plus slides (VWR International, Radnor, PA), and stored at room temperature until further use. After deparaffination by dipping the sections in xylene (VWR International) for 2× 10 minutes, the sections were rehydrated in graded ethanol with a final 2× 5 minutes step in distilled water. Epitope retrieval was performed in 10 mM sodium citrate solution (Sigma, St. Louis, MO) with 0.05% Tween 20 (Millipore, Burlington, MA), pH 6.0, by heating the sections in a steamer at 96°C for 20 minutes. Sections were washed twice in phosphate buffer ([PB]: 0.05 mM Na_2_HPO_4_·2H_2_O, 0.05 mM NaH_2_PO_4_·H_2_O, pH 7.4). PB with 10% normal donkey serum (Gentex, Zeeland, MI) and 0.4% Triton-X 100 (Roche Diagnostics, Basel, Switzerland) was applied at room temperature for 1 hour to permeabilize the tissue and to block nonspecific binding of the primary antibodies. Primary antibodies were diluted in PB with 0.4% Triton-X 100 and 3% normal donkey serum (Supplementary Data  Table S2). The incubation was performed in a humidified chamber at room temperature overnight, followed by washing in PB before secondary antibodies were applied. Secondary antibodies were incubated at 10 µg/mL in PB at room temperature for 1.5 hours. Nuclei were stained with Hoechst (1:1000; Invitrogen H3569, Life Technologies, Waltham, MA). The sections were washed in PB, and to reduce autofluorescence the sections were incubated in Sudan Black (0.3% Sudan Black dissolved in 70% EtOH [Sigma]) for 7 minutes, and then washed with 70% EtOH for 1 minute. Finally, the sections were washed in PB and embedded in Mowiol (0.1 M Tris-HCl, pH 8.5, 25% Glycerol; Millipore), 10% w/v Mowiol 4-88 (EMD Millipore Chemicals).

#### Image acquisition and analysis

Images of human brain sections were taken at 3 randomly selected locations within the grey matter of 1 brain section of the medial frontal gyrus. At the time of imaging, the investigator was blinded for the experimental group (aSAH or control). The sections were imaged with a 20× magnification on an epifluorescence microscope (Zeiss AxioImager M2 fluorescent microscope with N-Achroplan objectives, AxioCam MRm camera and Software Zen 2011). Images were evaluated and analyzed with the program ImageJ 2.0 (NIH, Bethesda, MD). For the microglia analysis (Iba-1 staining), the images were binarized using the triangle auto threshold algorithm in ImageJ. The percentage of the total area covered by Iba-1-immunopositive staining was measured. Particles larger than 100 pixels were considered as single cells. To analyze the activation of astrocytes, the intensity of the glial fibrillary acidic protein (GFAP) immunostaining was quantified by calculating the mean intensity per image. Furthermore, the percentage area of the GFAP-immunopositive area was quantified.

### Microthrombosis and microglia surface area

#### Human postmortem brain tissue

In the frontal cortex and hippocampus of 8 aSAH patients, we assessed the correlation between microthrombosis and microglia by investigating microglia surface area around vessels with and without microthrombosis (Table 1). We excluded tissue of aSAH patients with a neurodegenerative disease or amyloid angiopathy based on medical and/or autopsy reports.

#### Immunohistochemistry

Sections of 4-μm were cut, mounted on slides, deparaffinized and rehydrated as described above. A triple staining was performed (Fibrinogen, Iba-1, and Vimentin) (Supplementary Data  Table S1). Fibrinogen was stained with a peroxidase coupled antibody and 3,3′‐diaminobenzidine (DAB) and Iba-1 and Vimentin were stained with fluorescent antibodies. First, sections were deparaffinized and endogenous peroxidase was removed by incubation with H_2_O_2_ in phosphate-buffered saline ([PBS]; 1:100) for 10 minutes. Antigen retrieval was performed by heating sections to 96°C in sodium citrate buffer (10 mM, pH 6.0) for 20 minutes. Sections were permeabilized and blocked with 0.1% bovine serum albumin (BSA), 1% normal horse serum (NHS), 0.2% Triton-X 100 in PBS for 30 minutes and incubated with a primary antibody (fibrinogen 1:1000, diluted in PBS with 0.1% BSA and 1% NHS) at 4°C overnight. The sections were then incubated with a secondary antibody (goat-anti-rabbit-biotin 1:400) for 30 minutes at room temperature and staining was revealed with an avidin-biotin (ABC) kit and DAB. Sections were permeabilized and blocked once more and incubated overnight at 4°C with primary antibodies (Iba1 goat 1:1000 and vimentin chicken 1:1000) after which they were incubated with secondary antibodies (anti-goat-Cy3 1:1400 and anti-chicken-AF488 1:1400) for 1 hour at room temperature. Nuclei were stained with Hoechst (1:1000 in PBS +0.1% BSA). Finally, sections were washed with PBS, embedded in Mowiol, and dried overnight at 4°C.

#### Image acquisition and analysis

Images were taken with a brightfield and epifluorescence microscope (×40 objective, Zeiss AxioImager) at 5 randomly selected locations in the grey matter of 1 brain section of the frontal cortex and hippocampus. Seven images were excluded in a later phase as these images were overexposed (Supplementary Data  Table S3). Image analysis was performed with ImageJ 2.1 (NIH). On each image, 1 vessel with microthrombosis and 1 without microthrombosis were selected manually based on the fibrinogen and vimentin staining. Isolated vessels were selected. Regions of interest were created for all selections and enlarged 3 times (standard enlargement of 50 pixels, 12.8 µm, scale 0.2560 µm/pixel): ring 1 (50 pixels, 0–12.8 µm from the selected vessel), ring 2 (50 pixels, 12.8–25.6 µm from the selected vessel), and ring 3 (50 pixels, 25.6–38.4 µm from the selected vessel). Images were then binarized using the triangle auto threshold algorithm in ImageJ. The microglia surface area was measured by the percentage of Iba-1-immunopositive staining and analyzed for each area and the mean of all areas.

### Statistical analysis

To analyze microglia and astrocyte response after aSAH, a Student t-test was performed. A mixed model analysis was used to analyze microglia surface area around vessels with and without microthrombosis for each ring and the mean of all rings. We included a random intercept to correct for multiple measurements per patient and an unstructured covariance matrix to correct for associations between measurements of the rings. Models were estimated under restricted maximum likelihood and the validity of the model assumptions (normality, homoscedasticity) was assessed with an analysis of residuals. From the mixed model analysis, we derived estimated marginal means with 95% confidence intervals and p values for the differences between means. The datasets generated during the current study are available from the corresponding author on reasonable request.

## RESULTS

### Microglia and astrocyte response

**Figure 1.**
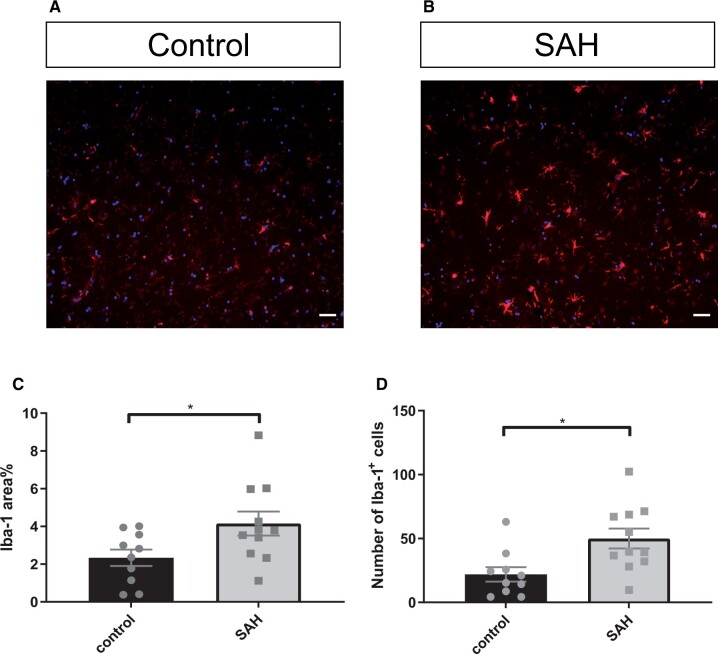
Microglial response in the frontal cortex of aSAH patients. **(A**, **B)** Representative images of Iba-1-positive cells in the frontal cortex of a control donor (**A**), and an aSAH patient **(B)**. **(C)** Iba-1 area% is increased after human aSAH. **(**D**)** The number of Iba1-positive cells is increased after aSAH. Scale bars: 40 µm, points in graphs represent individuals; Student t-tests; *p ≤ 0.05; **p ≤ 0.01; Bars and error bars in mean ± SEM; aSAH: aneurysmal subarachnoid hemorrhage.

**Figure 2.**
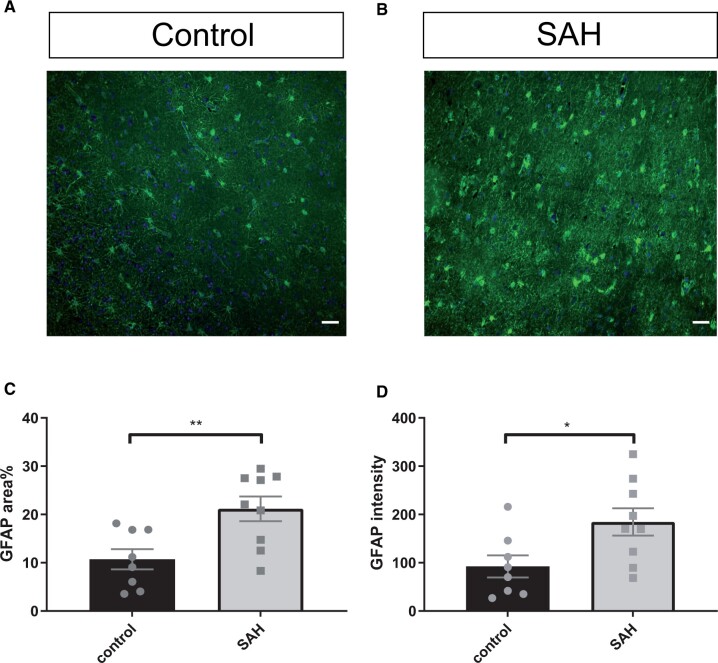
Astrocyte response in the frontal cortex of aSAH patients*.* **(A, B)** Representative image of GFAP-positive cells in the frontal cortex of a control **(A)**, and of an aSAH patient **(B)**. **(C)** GFAP area% is increased after aSAH. **(D)** The intensity of GFAP is also increased after aSAH. Scale bars: 40 µm; points in graphs represent individuals; Student t-tests; *p ≤ 0.05; **p ≤ 0.01; Bars and error bars in mean ± SEM; aSAH: aneurysmal subarachnoid hemorrhage.

A total of 63 measurements on 21 tissue sections (11 from aSAH patients and 10 from controls) were performed. The number of Iba-1-positive microglia per 0.0026 mm³ was 50 ± 8 in aSAH patients and 20 ± 5 in controls, p < 0.01 (mean ± SEM; Fig. 1). Iba-1 surface area (%) was 4.2 ± 0.6 in aSAH patients and 2.2 ± 0.4 in controls, p < 0.05 (mean ± SEM; Fig. 1). The surface area (%) of the astrocytic intermediate filament protein GFAP immunostaining was 21.2 ± 2.6 in aSAH patients and 10.7 ± 2.1 in controls, p < 0.01 (mean ± SEM; Fig. 2). The intensity of GFAP immunostaining was 184 ± 28 (arbitrary units) in aSAH patients and 92 ± 23, p < 0.05 in controls (mean ± SEM; Fig. 2).

### Microthrombosis and microglia surface area

**Figure 3.**
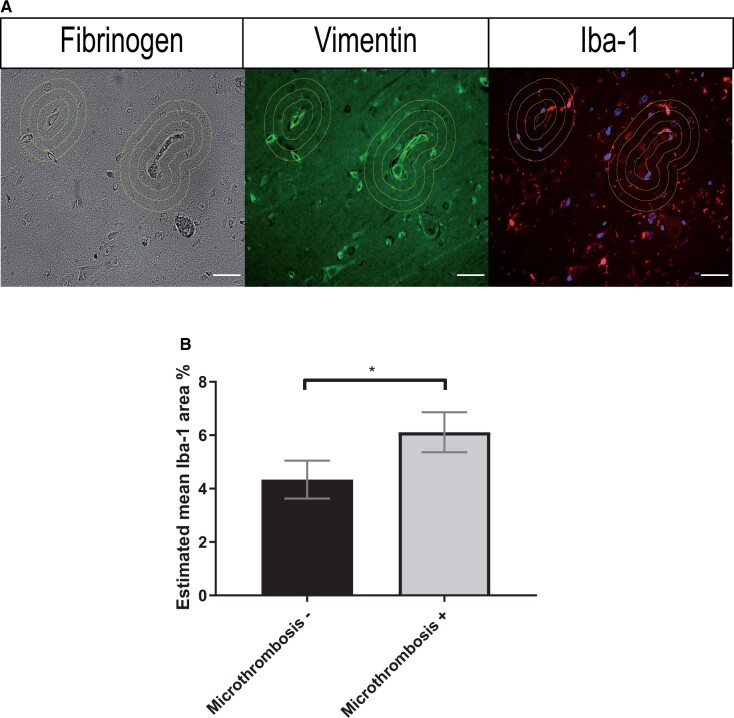
Microthrombosis and microglial response in the hippocampal and frontal cortex of aSAH patients. **(A)** Representative images of fibrinogen, vimentin, and Iba-1 staining with the enlarged selections of a vessel without and with microthrombosis. The circles indicate the areas in which we quantified glial activation; **(B)** Estimated mean Iba-1 surface area percentage is higher around vessels with microthrombosis compared to vessels without microthrombosis in brain tissue from aSAH patients. Scale bars: 40 µm; Mixed model; **p ≤ 0.01; Bars and error bars in estimated mean ± SEM; aSAH: aneurysmal subarachnoid hemorrhage; Microthrombosis−: vessel without microthrombosis; Microthrombosis+: vessel with microthrombosis.

We analyzed 126 measurements on 14 tissue sections of 8 aSAH patients. For the mean of all rings (0–38.4 µm from the vessel), the microglia surface area (%) was 6.1 [5.4–6.9] for vessels with microthrombosis and 4.3 [3.6–5.0] for vessels without microthrombosis, p < 0.001 (estimated marginal means with [95% CI]; Fig. 3). For each of the rings, the microglia surface area was higher around vessels with microthrombosis than around vessels without microthrombosis, with a statistically significant difference between ring 1 (0–12.8 µm) and 3 (25.6–38.4 µm) (Supplementary Data  Table S4).

## DISCUSSION

We showed that the number and surface area of microglia is twice as high in postmortem brain tissue of patients who died after aSAH compared to controls. The increase in number and surface area suggests activation of microglia. Astrocytes showed a clear increase in GFAP intensity and surface area, a marker for astrocyte reactivity. We also found that the microglia surface area was approximately 40% larger around vessels with microthrombosis than around vessels without microthrombosis. This finding shows that microthrombosis and microglia are interrelated.

In prospective cohort studies, increased levels of astrocytic markers GFAP and S100b were found in serum of aSAH patients during the first 2 weeks after ictus (19, 20). Maximum GFAP and S100b serum levels correlated with clinical outcome (19, 21). However, these findings may represent just brain injury instead of astrocyte activation, because GFAP and S100b are released into the serum upon glial cell death. A study on human SAH brain tissue found an increase in AQP4 expression, a water channel highly expressed by astrocytes, compared to controls (22). This finding suggests astrocytes change at a molecular level after aSAH but does not answer the question whether astrocytes become reactive. Our study now shows that astrocytes indeed are activated in postmortem brain tissue of aSAH patients. This finding is important because astrocytes may contribute to brain injury upon activation (10).

In aSAH patients, we could not find any study in the literature on the correlation between microthrombosis and neuroinflammation. Experimental studies on animal models for multiple sclerosis and Alzheimer disease show that fibrinogen leakage from the vasculature, due to a disturbed blood–brain barrier, induced perivascular microglia activation and clustering (23–25). Inhibition of fibrin formation or the fibrinogen binding motif reduces neuroinflammation, axonal damage, and synaptic deficits in these studies. A disturbed blood–brain barrier has also been observed in aSAH patients (26). We postulate a mechanism in aSAH similar to that in multiple sclerosis and Alzheimer disease, in which fibrinogen leakage from vessels with microthrombosis results in microglia activation and clustering. Since microthrombosis probably plays a role in the pathogenesis of both early brain injury and delayed cerebral ischemia after aSAH, we hypothesize that parenchymal gliosis is both part of a global ischemic event (resulting from an acute decrease in cerebral perfusion during bleeding of the aneurysm), and the result of delayed cerebral ischemia, which is mostly more focal. Conversely, it can be that blood breakdown products in the subarachnoid space lead to neuroinflammation, which in turn promotes the formation of microthrombi.

The astrocytic response has not been studied before in aSAH human postmortem brain tissue. This probably results from the very limited availability of such tissue samples. Autopsy is often not done in aSAH patients because the cause of death is known from premortem brain imaging. Instead, the glial response has been extensively studied in experimental models of SAH. These studies showed diffuse microglial and astrocyte activation after experimental SAH (6, 10, 11, 12). Following SAH, the morphology of microglia changes to an activated state, as their processes become shorter. In response to the hemorrhage, activated microglia will phagocytose blood components and release pro-inflammatory cytokines to induce an immune response (10). Inhibition of microglia activation reduced neuronal cell death in experimental SAH (6), but the exact mechanism of neuronal injury is unknown. Astrocytes respond to SAH in experimental studies by a multicellular process called reactive gliosis, which in turn can lead to the formation of scar tissue in the brain with both beneficial as well as detrimental effects (10, 17). Furthermore, activated astrocytes are involved in blood–brain disruption, the production of reactive oxygen species, and cell death after experimental aSAH (10–12).

Several limitations of this study need to be mentioned. First, our sample consists of a group of patients who had died at different timepoints after aSAH. The glial cell response and microthrombosis burden differ depending on the timepoint of death: the numbers of microglia are highest between days 5–15 after aSAH (6), while microclot burden is highest during the first days after the hemorrhage (15). Since most patients in our study on the correlation between microthrombosis and microglia surface area died in the first few days after aSAH, we expect that the majority of our sample had a high microclot burden and still a relatively low number of microglia. This could mean that the difference in microglia surface area around vessels with and without microthrombosis is less pronounced in our sample than in patients who die at a later timepoint. Second, since this is a postmortem study, all aSAH patients died from the hemorrhage, which means our sample may not be representative for aSAH patients with a more favorable outcome. However, since aSAH patients who survive often develop brain injury, the underlying processes are presumably similar to patients who died from aSAH, although the response may be less pronounced. Third, although controls were selected for their non-neuropathological cause of death, we could not rule out deterioration of brain tissue, either upon death or between the time of death and the fixation process. However, these conditions presumably would apply to both groups, resulting in comparable deterioration. We therefore assume the differences we found in glial response are due to an aSAH. Fourth, as a marker for microglial surface area, we used Iba-1 immunostaining, which is a microglial and macrophage-specific calcium-binding protein. Therefore, it does not differentiate between microglia and macrophages. Nevertheless, previous research has shown that the glial response in the brain of aSAH patients is mostly orchestrated by microglia (6). Moreover, although an increase in Iba-1 surface area suggests an activated state of microglia, it should be combined with more specific histological markers such as the major histocompatibility complex-II (MHCII) and CD68 to further investigate microglial activation (27). Microglia phenotype change is disease- and context-dependent; therefore, more in depth molecular phenotyping is needed to determine the exact phenotype changes in aSAH (28). Fifth, measurements for this study were performed on 2D histological sections. Ideally, one would perform a 3D histological evaluation, but this was not possible with the available material. Cell structures beneath or above the area of the sections could have affected our results. Also, due to the 2D nature of our samples, shapes of the vessels differed between measurements. We would expect that with the large number of measurements such an effect would influence the results in aSAH patients and controls and vessels with and without microthrombosis evenly. However, our results first have to be confirmed in 3D histological sections before a definite correlation between microglia and microthrombosis can be established. Because vessels with and without microthrombosis had to be manually selected, it was not possible to blind the investigator for this part of the study. Selection and observer bias could therefore have affected our results. Lastly, although our findings show a correlation between microthrombosis and microglia, the nature of this correlation and whether it is bidirectional cannot be deferred from our results.

In conclusion, our study found increased surface area of microglia and astrocytes after aSAH and a correlation between microthrombosis and microglia. Further research is needed to investigate whether the prevention of microthrombosis results in decreased neuroinflammation, which may decrease brain injury and thereby improve the prognosis for aSAH patients.

## Supplementary Material

nlad050_Supplementary_Data
