# Supplementary material for: Glial cell response and microthrombosis in aneurysmal subarachnoid hemorrhage patients: An autopsy study
Source: J Neuropathol Exp Neurol. 2023 Jul 21;82(9):798–805. doi: 10.1093/jnen/nlad050 (PMC10440719; doi:10.1093/jnen/nlad050)
Supplement: nlad050_Supplementary_Data [file nlad050_supplementary_data.docx]

**Supplementary Material**

**Glial cell response and microthrombosis in aneurysmal subarachnoid hemorrhage patients: an autopsy study**Email corresponding author: i.koopman-4@umcutrecht.nl

**Supplementary Table S1. Sample characteristics**

| **Microglia and astrocyte response** | | | | |
| --- | --- | --- | --- | --- |
| **Sample** | | **Location of ruptured aneurysm** | **Cerebral infarction according to autopsy report (excluding global ischemic changes)** | |
| 1 | | Acom | Bifrontal | |
| 2 | | Acom | Parieto-occipital right lobe | |
| 3 | | Left Acom and left MCA | Left basofrontal and temporal lobe | |
| 4 | | Acom | Brainstem | |
| 5 | | BA | No cerebral infarction | |
| 6 | | Acom | Neocortex | |
| 7 | | Left ICA | Hippocampus | |
| 8 | | Right PA | No cerebral infarction | |
| 9 | | Acom | Left occipital lobe | |
| 10 | | Left MCA | No cerebral infarction | |
| 11 | | Acom | Left frontal lobe | |
| **Microthrombosis and microglia surface area** | | | | |
| **Sample** | **Aneurysm location*** | | | **Cerebral infarction territory according to autopsy report (excluding global ischemic changes)** |
| 1 | Right MCA | | | No cerebral infarction |
| 2 | Acom and BA | | | No cerebral infarction |
| 3 | Right VA and Left ICA | | | Left frontal lobe, watershed infarction Acom and MCA right and left hemisphere |
| 4 | Left ACM | | | Left MCA |
| 5 | Left ACM | | | No cerebral infarction |
| 6 | BA | | | Thalamus right and left hemisphere and brain stem |
| 7 | Left Pcom | | | MCA left and right hemisphere |
| 8 | Right ICA | | | MCA right hemisphere |

VA: vertebral artery; BA: basilar artery; Pcom: posterior communicating artery; Acom: anterior communicating artery; MCA: Middle cerebral artery; ICA: internal carotid artery; PA: Pericallosal artery.

**Supplementary Table S2**. Primary antibody characteristics

| **Antibody** | **Host** | **Catalog number** | **Manufacturer** | **Dilution** |
| --- | --- | --- | --- | --- |
| **Microglia and astrocyte response** | | | | |
| Iba1 | *Rabbit polyclonal* | 019-19741 | Wako | *1:2000* |
| GFAP-pan | *Rabbit polyclonal* | Z0334 | Dako | *1:2000* |
| **Microthrombosis and microglia surface area** | | | | |
| Fibrinogen | *Rabbit anti-Fibrinogen* | 134387 | DivBio | *1:1000* |
| Iba1 | *Goat polyclonal* | AB5076 | Abcam | *1:1000* |
| Vimentin | *Chicken* | AB5733 | MilliporeSigma | *1:1000* |

**Supplementary Table S3**. Microthrombosis and microglia surface area: sections imaged and images

| **Case** | **Section analyzed** | **Images analyzed** |
| --- | --- | --- |
| 1 | Frontal cortex and hippocampus | 7 (3 images of the frontal cortex missing) |
| 2 | Hippocampus | 4 (1 image of the hippocampus missing) |
| 3 | Frontal cortex and hippocampus | 10 |
| 4 | Frontal cortex | 5 |
| 5 | Frontal cortex and hippocampus | 10 |
| 6 | Frontal cortex and hippocampus | 10 |
| 7 | Frontal cortex and hippocampus | 10 |
| 8 | Frontal cortex and hippocampus | 7 (3 images of the hippocampus missing) |
| Total analyzed | | 63 images |

**Supplementary Table S4**. Results of microthrombosis and microglia immunohistochemistry in human aSAH brain tissue: each ring separately

| **Measure** | **Ring** | **Group** | **Estimated marginal means and 95% CI** | **p value differences**  **between means *** |
| --- | --- | --- | --- | --- |
| Human Iba-1 area (%) | Ring 1 | Microthrombosis – | 4.8 (3.9-5.7) | 0.02 |
|  |  | Microthrombosis + | 6.4 (5.4-7.5) |  |
|  | Ring 2 | Microthrombosis – | 4.5 (3.6-5.5) | 0.07 |
|  |  | Microthrombosis + | 5.5 (4.7-6.3) |  |
|  | Ring 3 | Microthrombosis – | 3.7 (2.9-4.4) | <0.001 |
|  |  | Microthrombosis + | 6.4 (5.6-7.3) |  |

*Bonferroni adjustment for multiple comparison; CI: 95% confidence interval; aSAH: aneurysmal subarachnoid hemorrhage; Microthrombosis -: vessel without microthrombosis; Microthrombosis +: vessel with microthrombosis.
